# Impact of the Lymphatic Filariasis Control Program towards elimination of filariasis in Vanuatu, 1997–2006

**DOI:** 10.1186/s41182-017-0047-8

**Published:** 2017-06-01

**Authors:** Tammy Allen, Fasihah Taleo, Patricia M. Graves, Peter Wood, George Taleo, Margaret C. Baker, Mark Bradley, Kazuyo Ichimori

**Affiliations:** 1 College of Public Health, Medical and Veterinary Sciences, James Cook University, Cairns, Queensland Australia; 2 Vanuatu Ministry of Health, Government of the Republic of Vanuatu, Port Vila, Vanuatu; 3 James Cook University, Cairns, Queensland Australia; 4 RTI International, Washington District of Columbia, USA; 5 Global Health Program, GlaxoSmithKline (GSK), Brentford, UK; 6 Nagasaki University, Nagasaki, Japan

**Keywords:** Vanuatu, Lymphatic filariasis, PacELF, Elimination, Mass drug administration

## Abstract

**Background:**

Lymphatic filariasis (LF) occurs when filarial parasites are transmitted to humans through mosquitoes. The filarial worms affect the lymphatic system which leads to abnormal enlargement of body parts, chronic pain, disability, and social discrimination. In 1999, a commitment was made to eliminate LF from the Pacific Region by 2010. The Pacific Program to Eliminate LF began, with Vanuatu being one of the 16 endemic countries included in this program.

**Methods:**

In 1997/1998 a LF prevalence baseline survey was conducted to determine the need for mass drug administration (MDA) in Vanuatu. In 1999, the Vanuatu Lymphatic Filariasis Control Program was established, and nationwide MDA was implemented from 2000 to 2004. LF prevalence was collected during the MDA through sentinel site and spot check surveys, and after 5 years of MDA. MDA implementation methods included health worker training, social mobilization, and culturally appropriate health promotion strategies.

**Results:**

LF prevalence at baseline was 4.79%; after MDA this declined to 0.16% in 2005/2006. Average MDA coverage ranged from 75.5–81.5% across 5 years. All three evaluation units surveyed in 2005/2006 were below the 1% threshold required to stop MDA.

**Conclusions:**

The LF Control Program between 1997 and 2006 was successful in reducing LF prevalence to <1%. High MDA coverage was a critical component of this success. This period of the Vanuatu LF Control Program played an important role in helping to eliminate LF in Vanuatu.

## Background

Lymphatic filariasis (LF) occurs when filarial parasites are transmitted to humans through mosquitoes which pick up the microfilarial stage from the peripheral blood. The adult filarial worms reside in, and affect, the lymphatic system and can result in abnormal enlargement of body parts, chronic pain, disability, and social stigmatization [1]. In 2015, LF was endemic in 55 countries [2]. There continues to be an estimated 67.88 million cases of LF worldwide, including 36.45 million microfilaria (Mf) carriers, 19.43 million hydrocele cases, and over 16.68 million cases of lymphoedema or elephantiasis [3].

### History of LF in the Pacific

**Fig. 1:**
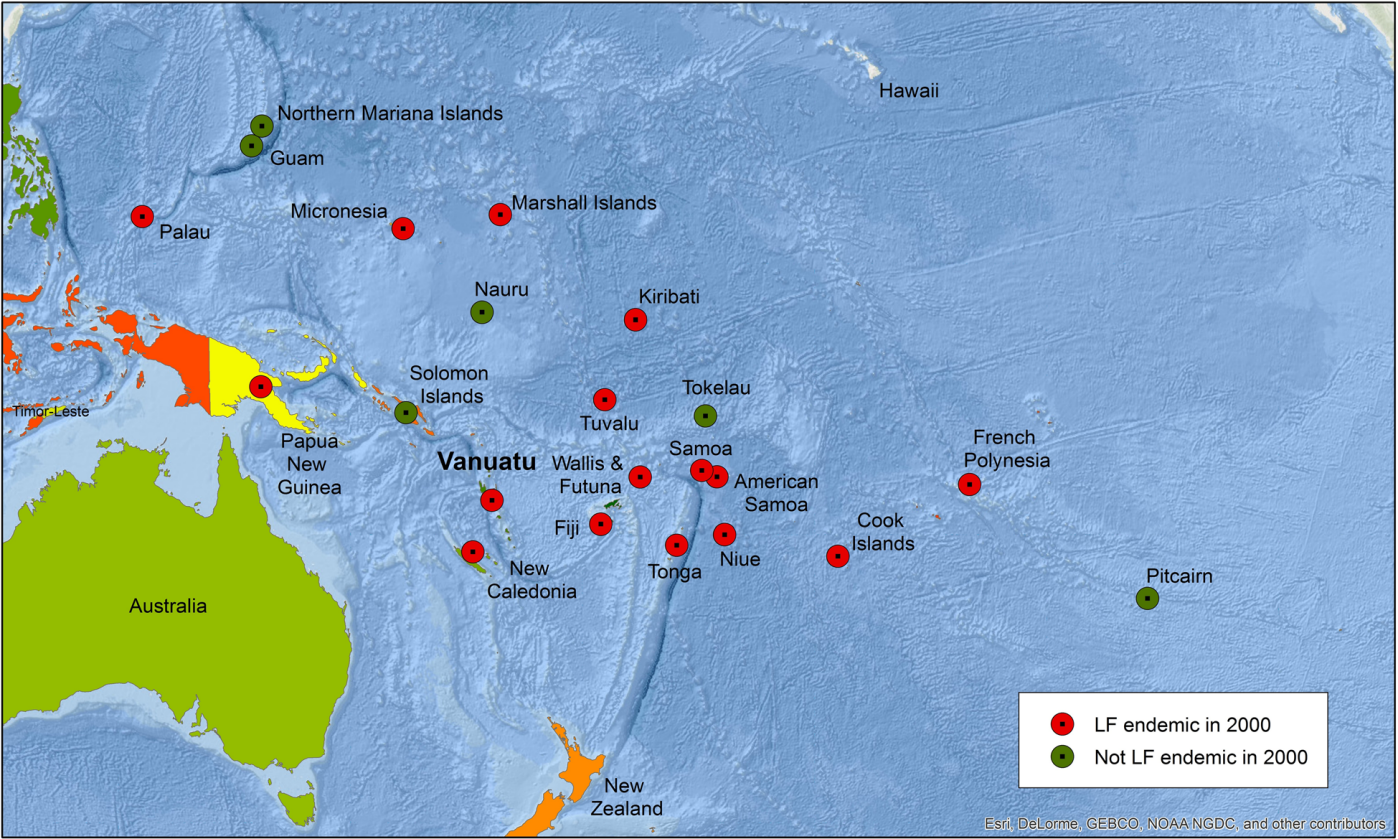
LF endemicity in the Pacific in 2000

LF is a significant public health issue in the Pacific Region [4]. In 2000, 16 of the 22 Pacific countries or territories were classified as endemic and in need of interventions to eliminate LF (Fig. 1) [5].

In 1999, the World Health Organization (WHO), Western Pacific Regional Office (WPRO), together with the Secretariat for the Pacific Community (SPC) and public health officials from Pacific Countries initiated the Pacific Program to Eliminate LF (PacELF) in Pacific Countries and Territories [6]. PacELF was a WHO led and supported program, based on Japan’s LF elimination strategy in the 1970s. PacELF focused on:

- A community-based and self-help driven approach
- Regional collaboration to benefit the entire Pacific family
- Pacific island country ownership
- Operational flexibility—each country chose its own mass drug treatment operational strategy, including customized social mobilization and information, education, and communication activities
- A simple core package of activities—provision and promotion of mass drug administration (MDA), morbidity treatment, and vector control including the use of mosquito nets when relevant
- Integration into existing health services [7].

### History of LF in Vanuatu

**Fig. 2:**
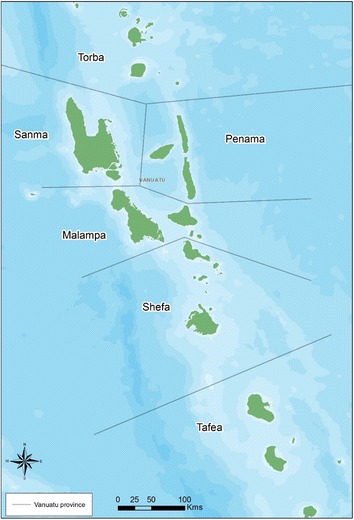
Vanuatu province boundaries

Vanuatu was one of the 16 endemic countries included in PacELF [8]. Vanuatu consists of an archipelago of 83 islands located between 12° to 21°S and 166^o^ to 171°E. The islands are divided into six provinces known from north to south as Torba, Sanma, Penama, Malampa, Shefa, and Tafea (Fig. 2). Based on the 2009 census the population of Vanuatu was 234,023. Mean population density was 19 persons per square kilometer, ranging from 11 in Sanma and Torba Province to 52 in Shefa Province [9].

Prevalence of LF in Vanuatu was first recorded in 1927. Buxton [10] reported results of a LF survey (*n* = 318 males tested over 12 years of age) in 16 islands of whom 100 (31%) were found to be Mf positive. Byrd and St Amant [11] reported surveys of Mf prevalence in 1943–1944 in 396 people (343 > 15 years and 53 < 15 years). Prevalence was 24.5% in adults and 3.8% in children. Males had a higher infection rate of 25.6% compared to 15.1% in females. Of those sampled, 183 were plantation workers who had lived in Espiritu Santo for <6 months but originated from 12 islands throughout Vanuatu. Among long-term residents, 33.6% were positive (*n* = 85 tested) in two villages on Espiritu Santo Island, and 0% (*n* = 104 tested) on two small islands offshore from Santo. A survey published in 1971 reported 32 cases of elephantiasis in a population of 2120 (1.5%) around Norsup in northern Malekula Island (Malampa Province), with the youngest case in a 14 year old [12]; the prevalence of microfilaria at night was 12.7% (*n* = 63). In 1978–1979, a survey of 7137 people on 12 islands from north to south found prevalence of 10% or above on the islands of Malekula and Ambrym (Malampa Province) and Epi (Shefa Province), 7% in Torres (Torba Province) and the remainder at 6% or below [13]. Elephantiasis cases (19 in total) were observed only on Banks, Santo, Maewo, and Efate Islands. MDA with Diethylcarbamazine (DEC) was given for 3 years in seven islands; Santo (Sanma Province), Hiu and Gaua (Torba Province), Malekula (Malampa Province), Erromango, Aniwa and Aneityum (Tafea Province), although details of the population numbers are not given [13]. In some areas MDA was combined with vector control (DDT spraying) or DDT spraying alone was done, and the reductions in prevalence and density of Mf were dramatic. However, elimination was not achieved, with transmission persisting on Malekula, Santo, Pentecost, and Epi Islands in the mid-1980s. The varying geographical prevalence of LF in Vanuatu is thus a result of environmental factors such as temperature variations and previous control methods on some islands.

There were no other significant LF prevalence studies conducted or programs implemented to specifically treat and eliminate LF in Vanuatu until 1997. There was however, a malaria control program in Vanuatu focused primarily on the use of insecticide-treated bed nets (ITN) to protect against mosquito bites. Given that the same species of mosquito *Anopheles spp.* transmits both malaria and LF in Vanuatu, this likely had some impact on LF prevalence.

In 1999, with PacELF support, the Vanuatu National LF Control Program was established within the Malaria and Other Vector Borne Diseases Control Unit (VBDCU) of the Vanuatu Ministry of Health (Ichimori K: Vanuatu national filariasis programme plan of work for 1998, unpublished). The program’s aim was to (1) assess the prevalence of filariasis in Vanuatu and treat positive cases and (2) eliminate the risk of LF to the population through MDA of albendazole (GSK Donation Program) and DEC, as well as vector control (WHO: Application from the ministry of Health of Vanuatu to support a national programme to eliminate lymphatic filariasis in 1998, unpublished).

This is the first published paper presenting comprehensive results of Vanuatu’s LF Control Program, building on Fraser et al.’s (2005) evaluation of mid-term results [14]. This paper describes LF prevalence before MDA, results from sentinel site and spot check surveys during MDA, and LF prevalence after 5 years of MDA. This paper also details the methods used to effectively deliver MDA. The post-MDA surveillance period and validation of elimination in 2016 through the Vanuatu Ministry of Health and PacELF efforts are described separately, together with morbidity surveys, in comparison papers.

## Methods

The aim of this paper is to evaluate the impact of Vanuatu’s LF Control Program on filariasis in Vanuatu from 1997 to 2006. Vanuatu’s LF Control Program consisted of three key phases, [6] namely:

1. Baseline mapping phase: the A survey of LF prevalence, conducted to determine the need for MDA
2. Intervention Phase which included:
  ◦MDA annually over 5 years
  ◦Health promotion through distribution of morbidity posters, morbidity treatment kits, promotion of mosquito net usage
  ◦The B survey of LF prevalence in specific sentinel sites, implemented after two MDA rounds
  ◦Periodic spot checks
3. Stop MDA Phase: the C survey of LF prevalence, to determine whether prevalence was below 1% for MDA to be stopped

### A survey (baseline survey)

The A survey was conducted in 1997/1998 to ascertain LF prevalence in Vanuatu. Samples were collected from persons >2 years of age in 51 villages throughout the 6 provinces of Vanuatu. The number of villages sampled per province was based on proportional population distribution by province from the 1989 National Census (Table 1).

**Table 1:** Proportion of population sampled

Province	Population per province^a^	% of total population	Total examined by province	% of total examined by province	No. of villages sampled per province
Torba	5985	4.2	227	4.4	4
Sanma	25542	17.9	833	16.3	7
Penama	22281	15.6	784	15.3	8
Malampa	28174	19.8	894	17.5	8
Shefa	38023	26.7	1429	27.9	14
Tafea	22414	15.7	952	18.6	10
Total	142419	100	5119	100	51

^a^1989 National Census Date Source: [16]

Convenience sampling was used at the village/household level, with residents (>2 years of age) of selected villages invited to participate. From the 51 villages, 4363 people were tested by Binax Immunochromatographic Test (ICT) to detect circulating antigen to *Wuchereria bancrofti* [6] and 4269 people were tested by blood (60 ul) slides at night to detect the presence of Mf. Blood slides were stained with Giemsa and read at the VBDCU in Port Vila. From the sample population, 3573 people received both ICT and Mf tests [15]. All antigen-positive and Mf-positive cases were treated with DEC+ albendazole [16].

### Mass drug administration

MDA is the recommended strategy required to suppress Mf numbers and transmission of the parasite over the life of the adult parasites, approximately 5 years [6]. The A survey in 1997/1998 demonstrated that LF prevalence was greater than 1% in Vanuatu. This level of prevalence qualified the country for free drug treatment, which was donated by WHO and GlaxoSmithKline to facilitate the elimination process. The Vanuatu Ministry of Health carried out five rounds of MDA nationwide from 2000 to 2004 [16]. MDA treatment included one dose of drugs (DEC and Albendazole) per person, per year using directly observed treatment (DOT). As weight was not always known, age was used to determine DEC dosage per person. 50 mg tablets were given as follows : 2–9 years of age, 2 tablets; 10–19 years of age, 5 tablets; 20–29 years of age, 7 tablets; 30–59 years of age, 8 tablets; >60 years, 7 tablets). Albendazole dosage was 400 mg per person. Pregnant women, those under 2 years and very sick people were excluded. MDA coverage of the national population was reported every year against the registered population, the eligible population, and the national population [6]. The latter denominator is used in this paper.

### MDA program implementation

#### Social mobilization and IEC (information, education and communication) strategy

The MDA communication strategy was based on a Knowledge, Attitudes and Practice (KAP) survey conducted a couple of months before the first round of MDA began. This helped to ascertain knowledge gaps on the disease and was used to inform the development of the IEC materials such as posters, leaflets and t-shirts. A second KAP survey was conducted after two MDA rounds to measure community opinion and levels of acceptance toward MDA to help inform future strategies.

Two training workshops were carried out prior to the MDA for nurses, nurse aides, and village chiefs. The aims of the workshops were to plan MDA dissemination strategies including social mobilization and communication strategies, prepare finances and conduct registration training.

Communication strategies began 2–3 weeks prior to each round of MDA to prepare the community for MDA. Working closely with the village chiefs, community leaders and church leaders was critical to the success of the program. In addition to including the village chiefs in the training workshops, province level staff organized official meetings with the chiefs using traditional custom approaches including ceremonies, drinking the local kava drink, killing and eating pigs, and gifting of mats. Community engagement in the weeks leading up to MDA included community talks, distribution of information leaflets, radio drama, and Q&A sessions with experts. Pictures were found to have a strong impact, so posters were developed with pictures of persons with signs and symptoms of LF.

During MDA, all the health workers continuously followed-up on any refusal cases and tried their best to address this to ensure high coverage. Melanesian culture hold health workers and community leaders in high esteem, and this assisted to optimize community acceptance towards MDA.

#### Drug supply chain, storage, and distribution

The donated drugs were ordered through WHO, were coordinated by the PacELF office, and were shipped to Vanuatu. Customs processing and storage fees were paid by the program to customs. Everything was pre-packed into health zones packages at national level and then distributed by the program. As the drugs did not require refrigeration, they were stored in government medical stores and health centers until distributed by nurses (as part of their community outreach role) and nurse aides. While the nurses distributed the drugs, the nurse aides supported the social mobilization efforts in the community and registration of treatment during the MDA. About 200 nurses took part in the annual MDA with 750 persons on average treated by each nurse, working over a 2–3-month period. 1–2 nurses would remain in clinics to see the normal patient load. MDA was door to door or office to office. In addition, children were treated in schools and in the community, and fixed posts were set-up over a period of 2 weeks after MDA. DOT was always enforced with provision of cups and water. Supervisors emphasized strict adherence to the DOT protocol. Travel allowance (1700 vatu, approximately USD15, the standard government rate) was given to nurses and nurse aides if they had to sleep overnight, and accommodation and transport costs were paid. In the urban setting of Port Vila, public health officials and health workers delivered the treatments with volunteers supporting registration and recordings. All unused drugs were returned to province level and stored in pharmacies for routine treatment and use in surveys.

#### Supervision

Supervision was led by the province level malaria supervisors and other public health provincial level managers. The supervisor’s role was to check in with each MDA team every 2–3 days. Supervisors would carry extra registration books and medicines, follow-up on any refusals, and compare numbers treated to the target population.

#### Adverse events

Communication included information on what to expect in terms of minor side effects. To address potential serious adverse events (SAEs), forms were distributed every year to province level. Only one case was ever reported, but was found not to be linked to treatment.

#### Program monitoring

Coverage was the key indicator calculated every year. Treatments were recorded in registration books which were shipped back to national level after each MDA. Books were numbered with a special code and receipt of registration books was carefully monitored, including tracking down of missing books. Front page summary sheets allowed for quick calculation of coverage rates and then all data was entered into a database. A coverage survey conducted after two rounds of MDA validated the quality of routinely reported data.

#### Vector control

During MDA, the VBDCU also actively engaged in malaria control programs including distribution of ITNs, education on the use of nets, and indoor residual spraying [17].

### B survey (sentinel site survey) and spot check surveys

During the MDA period, LF prevalence was monitored using ICT blood tests and Mf night blood slides at sentinel sites (B survey) and spot check sites.

#### B survey

Sentinel sites were chosen from A survey results, whereby the two villages with the highest antigen prevalence within the four highest endemic provinces were selected. These sites were Sola and Mosina Village (Torba Province), Sakau and Wanur villages (Penama Province), Orap and Unmet villages (Malampa Province), and Port Resolution and South River villages (Tafea Province) [14]. In 2002, after two rounds of MDA, the B survey was conducted to evaluate whether MDA was decreasing antigen prevalence in these villages of high endemicity [14]. All individuals >10 years were invited to have an ICT blood test (except in Port Resolution where only 300 were examined). Night blood slides for Mf were taken from antigen-positive persons only. Slides were prepared with 60 ul of blood, dried, stained with Giemsa, and examined at the VBDCU in Port Vila. Sentinel sites were surveyed again in 2005/2006, except for Malampa Province.

Along with the ICT blood test, individuals were also asked about their mosquito net usage. The following two questions were asked “do you usually use a bednet?” and “did you use a bed net last night?”.

#### Spot check surveys

As sentinel sites may receive more programmatic attention than other sites, additional spot checks were conducted to determine if further rounds of MDA were needed at certain villages or islands. In 2002, spot check surveys were conducted in persons >10 years in Redcliffe village (Penama) and Lingarak village (Malampa). Spot checks were also conducted at Vila Central Hospital (Shefa) and Santo Northern District Hospital (Sanma) for people receiving malaria slide tests. In 2003, spot checks were conducted in Lolowai Hospital (Penama) and Norsup Hospital (Malampa), and repeated again in Vila Central Hospital and Santo Northern District Hospital. In 2004, spot checks were conducted in the villages of North Ambrym (Malampa).

### C survey (transmission assessment survey or TAS 1)

In 2005 and 2006, after five rounds of MDA, the C survey was conducted in villages from three evaluation units (EU) created from Vanuatu’s six provinces. These EUs were (1) Torba, Sanma and Malampa; (2) Penama and (3) Shefa and Tafea (note: the urban areas of Luganville, Santo Island, Sanma Province, EU1 and Port Vila, Efate Island, Shefa Province, EU3, were excluded from the sampling frame). Combination of provinces to EUs and sampling frame were determined based on baseline antigen prevalence [18]. If antigen prevalence was found to be <1% (upper 95% confidence limit <2%) in each EU, then MDA could be stopped.

C survey sampling used systematic selection of clusters with probability proportional to village size through a method modified from UNICEF’s multiple indicator cluster survey design [19]. Target sample per EU was 30 clusters of 15 households (i.e. 450 households with estimated 2700 persons at 6 per household). Total target national sample was 90 clusters i.e. 1350 households or ~7100 persons. Sample clusters were systematically selected by a random start from villages listed with their number of clusters estimated from village size using census information. Cluster size of 15 households was chosen to (1) keep overall numbers of persons tested within reasonable limits for a one-day visit and (2) prevent the need to combine villages together due to many villages being quite small, but with large households. Respondents were tested for antigen prevalence through ICT and were asked about mosquito net usage. Night blood slides for Mf were taken from antigen-positive persons only. Persons ages 1 year or older were eligible to participate. The C survey also collected information about mosquito net availability and use for each participant.

Statistical analysis was carried out using Microsoft Excel and STATA 14. Data on participant characteristics were analyzed using descriptive statistics. Comparisons involving categorical variables were conducted with chi-squared tests, and exact binomial confidence intervals were used to analyze proportional statistics.

## Results

### A survey

A survey data was collected in 1997/1998. A total of 4363 ICT samples and 4330 Mf samples were collected from persons of all ages in 51 villages throughout the six provinces of Vanuatu. 4.8% of ICT samples were antigen-positive, and 2.5% of Mf samples were Mf-positive. Of the 3573 people who had both the ICT and Mf test, 5.8% were ICT positive, and 3.2% were Mf positive [15]. Six Mf positive cases were ICT negative.

Penama Province had the highest antigen prevalence with 14.9%, followed by Torba Province with 10.2%, Malampa Province at 7.1% and Tafea Province 2.5%. Shefa Province and Sanma Province had low levels of antigen positives: 0.1 and 0.3%, respectively. Prevalence of Mf was generally two to three times lower than antigen prevalence (Table 2).

**Table 2:** Antigen-positive and Mf positives—provincial level in 1997/98

Province	Total ICT tested	Antigen positive %	Total Mf slide tested	Mf Positive %	Total Tested both ICT and Mf slide	Antigen positive %	Mf positive %
Torba	59	10.2	227	3.1	59	10.2	5.1
Sanma	371	0.3	833	0.2	370	0.3	0
Penama	776	14.9	723	7.9	776	15.0	9.2
Malampa	846	7.1	894	3.6	846	7.1	3.8
Shefa	1363	0.1	640	0.2	574	0.2	0.2
Tafea	948	2.5	952	0.7	948	2.5	0.7
Total	4363	4.8	4269	2.5	3573	5.8	3.2

Data source: [16]

**Fig. 3:**
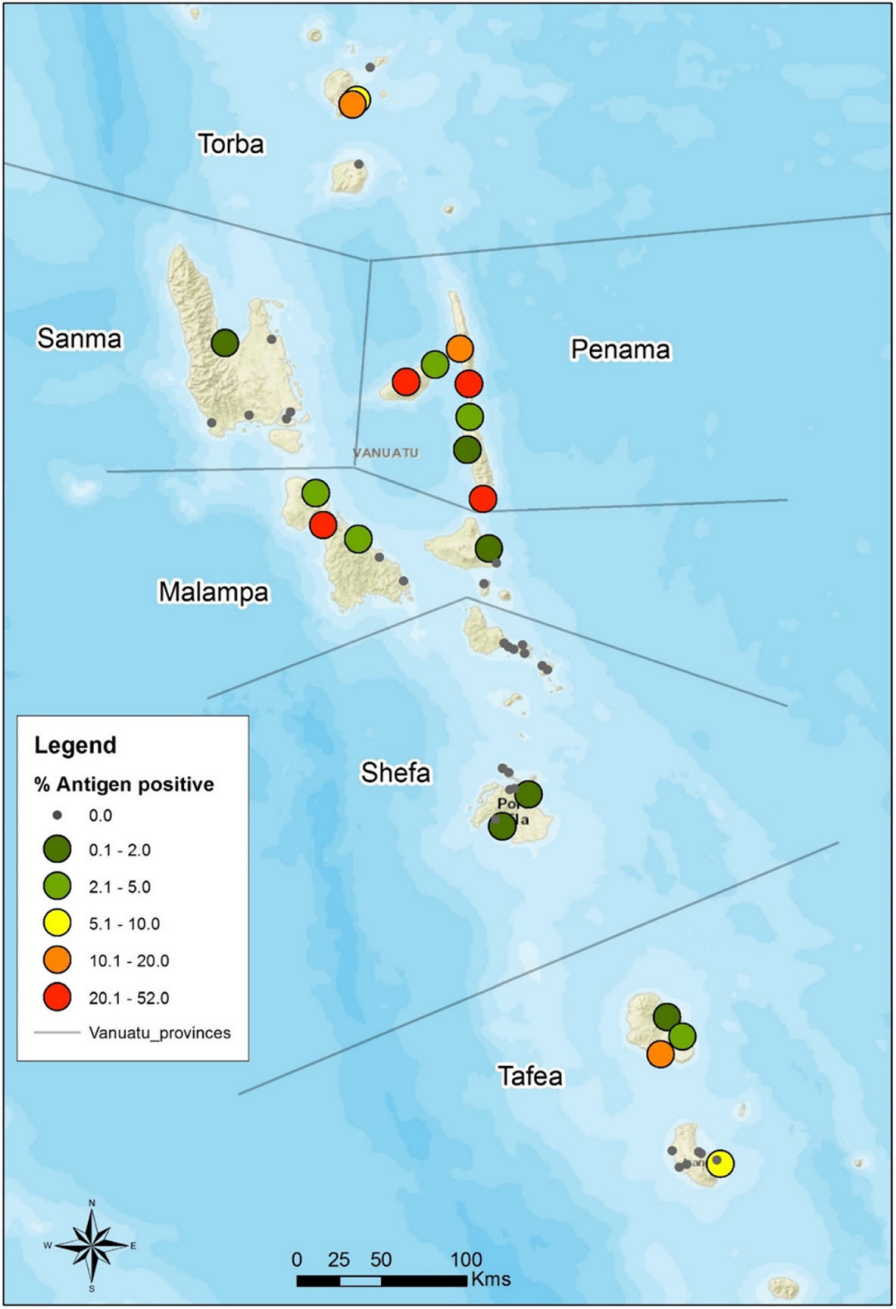
Antigen prevalence in Vanuatu villages 1997/1998

The spatial distribution of antigen-positive cases in each village surveyed can be found below (Fig. 3). More than half of the 51 villages (*n* = 28) had no positive cases. There were eight villages with greater than 10% antigen prevalence: two villages in Torba Province, four in Penama Province, one in Malampa Province, and one in Tafea Province.

#### Age and gender

Age-specific antigen prevalence in 1997/1998 showed 5.19% of children aged 3 to 10 years were antigen positive (*n* = 212). Antigen prevalence in the over 50 age group was between 10 and 11.5% antigen-positive (*n* = 380) (Table 3). Gender-related antigen prevalence (Table 4) showed males with a significantly higher prevalence than females: 5.8% in males and 3.9% in females (chi-square = 8.14 *p* = 0.004).

**Table 3:** 1997/1998 age and antigen prevalence

Age group	No. negative	No. positive	positive (%)
3 to 10	201	11	5.19
11 to 15	346	11	3.08
16 to 20	702	21	2.90
21 to 30	1116	46	3.96
31 to 40	642	45	6.55
41 to 50	368	20	5.15
51 to 60	211	24	10.21
61 to 70	123	15	10.87
70+	46	6	11.54
Grand total	4154	209	4.8

No data (*n* = 208). Data source: [16]

**Table 4:** 1997/1998 gender and antigen prevalence

Gender	No. negative	No. positive	% positive (%)
F	2267	93	3.9
M	1887	116	5.8
Grand total	4154	209	4.8

No data (*n* = 208). Data source: [16]

### Mass drug administration

After the A survey, MDA was conducted across six provinces from 2000 to 2004. With the exception of Torba Province in 2002, province level treatment coverage of MDA was between 75.5 and 81.5% [18] (Table 5).

**Table 5:** MDA coverage by province and year

Province	MDA coverage				
	2000 (%)	2001 (%)	2002 (%)	2003 (%)	2004 (%)
Torba	80.0	82.8	56.2	86.1	84.4
Sanma	77.2	73.5	76.2	80.2	73.4
Penama	90.5	82.6	78.8	80.8	79.1
Malampa	88.4	84.5	85.5	77.2	81.0
Shefa	81.8	86.7	78.4	81.8	82.1
Tafea	70.5	69.1	72.7	76.7	65.7
Average coverage	81.5	80.3	78.2	79.6	75.5

Data source: [16]

Estimated census population [6] were used as denominators to avoid underestimating population coverage

### B survey and spot check surveys

#### Sentinel site survey

**Fig. 4:**
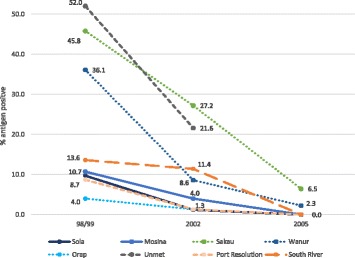
% antigen-positive for Vanuatu across eight sentinel sites from 1997/1998, 2002 and 2005/2006

In 2002, the B survey was conducted in Sola and Mosina (Torba Province), Sakau and Wanur (Penama Province), Orap and Unmet (Malampa Province), and Port Resolution and South River (Tafea Province). Survey results showed a decline in antigen prevalence in all sentinel sites compared to 1997/98. In 2005/2006, all sentinel sites except for Malampa Province were tested again. Antigen prevalence was 0% in all sites except for Sakau (6.4%) and Wanur (2.2%) (Penama Province). Overall, sentinel site results showed MDA was having an impact and decreasing antigen prevalence in high endemicity villages (Fig. 4). In terms of mosquito net usage, 75.5% of respondents answered “yes” when asked if they usually use a mosquito net (*n* = 1168). (note: there was high correlation between responses to “do you usually use a bednet?” and “did you use a bednet last night?” so it can be assumed that those who said that they “usually” use a bednet, slept in it the night before).

#### Spot check surveys

In 2002, spot check survey results showed Redcliffe Village (Penama) had 40% antigen-positive (*n* = 129 tested), Lingarak Village (Malampa) 0% antigen-positive (218 tested), Vila Central Hospital (Shefa) 3% antigen-positive (*n* = 254 tested), and Santo Northern District Hospital (Sanma) 2% antigen-positive (*n* = 168 tested).

In 2003, Santo Northern District Hospital showed 4.1% antigen-positive (*n* = 73 tested) and Vila Central Hospital 0% antigen-positive (*n* = 89 tested). Spot check surveys were also conducted in Lolowai Hospital (Penama) with 7.8% antigen-positive (*n* = 154 tested), and Norsup Hospital (Malampa) 13.5% antigen-positive (*n* = 74 tested). In 2004, spot check monitoring in the villages of North Ambrym (Malampa) found 19.2% antigen-positive (*n* = 551 tested). Based on this result, another three rounds of MDA were conducted in North Ambrym villages (1999 census population: 3899).

### C survey (TAS 1 survey)

In 2005/2006 C survey results in persons aged 1 year and above showed that antigen positive prevalence had dropped from 4.8% in 1997/1998 to 0.16% in 2005/2006 (0.274 upper 95% confidence limit) (Table 6). There were no Mf positive cases found.

**Table 6:** LF prevalence 1997/1998 and 2005/2006

Particulars	A survey 1997/1998	C survey 2005/2006
No. of villages tested	51	90
No. tested for antigen	4363	7657
No. antigen-positive	209 (4.79%)	12 (0.16%)
No. tested for Mf	4269	12^a^
No. Mf-positive	106 (2.48%)	0

^a^Note: only those antigen-positive were tested for Mf in 05/06

**Fig. 5:**
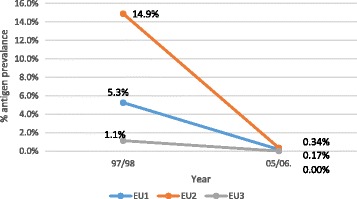
Evaluation units and antigen prevalence in 1997/98 and 2005/2006

C survey evaluation unit results are described below. In EU1, antigen prevalence was 0.17% (*n* = 2351 tested). EU2 had 0.34% antigen prevalence (*n* = 2353 tested), and EU3 had no antigen-positive cases (*n* = 2953 tested) (Table 7). For each EU, prevalence (and upper 95% confidence interval) had fallen to under the 1% threshold required to stop MDA. This reduction in antigen prevalence can be seen when comparing EU prevalence in 1997/1998 with 2005/2006 (Fig. 5).

**Table 7:** Antigen-positive prevalence–EU level in 2005/2006

(EU)	Province	Number tested	Antigen positive		Upper 95% binomial confidence interval %
			*n*	%	
1	Torba	236	4	1.69	
	Sanma	769	0	0.00	
	Malampa	1346	0	0.00	
	Total EU 1	2351	4	0.17	0.44
2	Penama	2353	8	0.34	
	Total EU 2	2353	8	0.34	0.67
3	Shefa	1689	0	0.00	
	Tafea	1254	0	0.00	
	Total EU 3	2953	0	0.00	0.13
Total population sampled		7657	12	0.16	0.27

Data source: [16]

#### Age and antigen prevalence

**Fig. 6:**
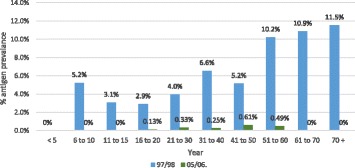
Age-specific antigen prevalence in 1997/1998 and 2005/2006

Age-specific antigen prevalence in 2005/2006 reduced in all age groups compared with 1997/1998. In 2005–2006, there were no antigen-positive cases under 5 years (*n* = 1166), 6–10 years (*n* = 1461) or 11–15 years (*n* = 1041). Antigen prevalence was below 0.61% for ages 21–60 years (*n* = 2919). There was no antigen-positive case for 61+ years (*n* = 298) (Fig. 6).

#### Gender and antigen prevalence

**Fig. 7:**
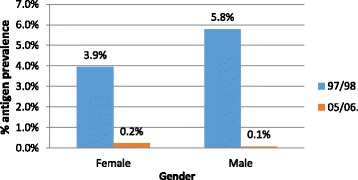
Gender-specific antigen prevalence in 1997/1998 and 2005/2006

Gender-specific prevalence in 2005/2006 showed no significant difference between male and female, with both genders showing a reduction to less than 0.3% in prevalence compared with 1997/1998 (Fig. 7).

#### Mosquito net use and availability

The C survey also collected information about mosquito net availability and use. The survey found 63% of respondents said they had a bed net, and most of these respondents reported sleeping under a net. The small number of positives in the C survey precluded further analysis of the relationship between antigen positivity and net use after MDA.

## Discussion

This study evaluated the impact of the LF control program in Vanuatu from 1997 to 2006. The A survey conducted in 1997/1998 showed prevalence of LF in Vanuatu was 4.79%. Notwithstanding limitations of convenience sampling within selected villages, the A survey showed that LF prevalence was more than the 1% required to justify the need for a specific LF control program. The A survey results reflected a typical age-specific prevalence curve and higher prevalence in males than females. Lower prevalence in females is a pattern that has been observed worldwide and may be due to differential exposure, increased resistance, or physiological factors [20]. The results of the A survey have been presented in summary form previously in program reports [6, 16], and a summary publication [21].

MDA was implemented in Vanuatu from 2000 to 2004 using a combination of DEC and Albendazole. Average MDA coverage ranged from 75.5 to 81.5% across the 5 years. The success of MDA is often measured by how effectively it is implemented on the ground. Factors integral to this include the dissemination of culturally appropriate, evidence-based community awareness strategies, development of trust, appropriate training, easy access to treatment through numerous channels, and quality data collection and analysis [22]. These were all the factors found to influence the high level of MDA coverage in Vanuatu. Specifically, the following strategies were seen as crucial elements:

1. Awareness strategies used before MDA, such as posters and leaflets with clear, visual messages on the need to take medicine, side effects, the disease and transmission
2. MDA being implemented by the health workers who had established relationships with their community and made sure to follow-up on non-treated cases
3. Engaging with community leaders to ensure culturally appropriate strategies that promoted medicine compliance.

The C survey followed the PacELF guidelines in place at the time [6]. The survey was conducted in all ages in clusters randomly selected as proportional to population size, with a target threshold of 1% MDA prevalence. In Vanuatu, because of the variable prevalence at the start of the program, there was a concern that parts of the country might not be ready to stop MDA in 2006. However, the results from the C survey demonstrated that the threshold of <1% antigen prevalence across each EU had been met. Although all EUs passed the C survey threshold, the program proactively targeted a remaining hotspot area of higher prevalence in North Ambrym in Malampa Province (part of EU1) through three additional rounds of targeted MDA after 2006.

Regarding age-specific prevalence, if transmission had been interrupted in Vanuatu one would expect the age-specific curve to shift to the right, showing persistence of infection in older persons infected some time ago, and no new infection in young people. There is some evidence of that in the C survey results, but overall too few positives in 2005/06 to compare age groups.

Vanuatu is fortunate in being located at the fringe of the range of *Anopheles* transmission; since this mosquito is also the vector of malaria, the efforts of the VBDCU have assisted in eliminating LF as well. This is also demonstrated by the high usage of mosquito nets as reported in both the B survey and C survey results.

The huge efforts of the LF Control Program and the MDA coverage report show that the program successfully achieved low prevalence in 2005/2006. Further study of areas of remaining transmission after MDA may be useful to the other countries in the region and will be reported separate to this paper.

## Conclusions

The LF Control Program during 1997–2006 demonstrated an important contribution towards LF elimination. This paper showed that MDA successfully suppressed LF antigen prevalence in Vanuatu to below 1% in 2006. Evaluation of the next stage of Vanuatu’s elimination program, the post MDA surveillance period after 2006, and documentation of governance, partnerships and community engagement will be reported in companion papers to provide further insight and learning from Vanuatu’s LF Control Program, as well as PacELF and their achievements

## Abbreviations

A survey: PacELF baseline mapping survey
B survey: PacELF equivalent of sentinel site survey
C survey: PacELF Stop MDA survey done in all ages. Revised version would be TAS 1, but that is done in children only.
D survey: PacELF’s equivalent to child transmission survey and TAS, but with different target threshold and sample size.
DEC: Diethylcarbamazine
DOT: Directly Observed Therapy
GPELF: Global Program to Eliminate LF
GSK: GlaxoSmithKline
ICT: Immunochromatographic Test
ITN: Insecticide-treated bed net
LF: Lymphatic filariasis
MDA: Mass drug administration
Mf: Microfilariae
PacELF: Pacific Program to Eliminate LF
TAS: Transmission assessment survey
VBDCU: Malaria and Other Vector Borne Disease Control Unit
WHO: World Health Organization
WPRO: WHO Western Pacific Regional Office

## References

[1] Lymphatic filariasis. Fact sheet No. 102 [http://www.who.int/mediacentre/factsheets/fs102/en/]. Accessed 5 Feb 2017.

[2] WHO (2016). Weekly Epidemiological Record.

[3] Ramaiah KD, Ottesen EA (2014). Progress and impact of 13 years of the global programme to eliminate lymphatic filariasis on reducing the burden of filarial disease. PLoS Negl Trop Dis.

[4] Ichimori K, Graves PM, Crump A (2007). Lymphatic filariasis elimination in the Pacific: PacELF replicating Japanese success. Trends Parasitol.

[5] Graves PM, Wood P, Bossin H, Loukas A (2016). Lymphatic filariasis in Oceania. Neglected Tropical Diseases of Oceania.

[6] WHO (2006). The PacELF way: towards the elimination of lymphatic filariasis from the Pacific, 1999–2005.

[7] Ichimori K, Graves PM, Crump A (2007). Lymphatic filariasis elimination in the Pacific: PacELF replicating Japanese success. Trends Parasitol.

[8] WHO (1999). Global filariasis elimination programme: Application from Vanuatu – feedback from WHO.

[9] Vanuatu-National-Statistics-Office (2009). National Census of Population and Housing. Vanuatu National Statistics Office. Ministry of Finance and Economic Management.

[10] Buxton PA. Malaria and filariasis in the New Hebrides. researches in Polynesia and melanesia. Appendix II Memoirs of the London School of Hygiene and Tropical Medicine; 1927. p. 225–37.

[11] Byrd EE, St Amant LS (1959). Studies on the epidemiology of filariasis on central and south Pacific islands. SPC Technical paper on 125.

[12] Lagraulet J, Bonnin P (1971). Lymphatic filariasis at Mellicolo (New Hebrides; preliminary survey). Bull Soc Pathol Exot Filiales.

[13] Bouree P, Sauvagnac B, Montaville B (1987). Lymphatic filariasis in Vanuatu. Bull Soc Pathol Exot Filiales.

[14] Fraser M, Taleo G, Taleo F, Yaviong J, Amos M, Babu M, Kalkoa M (2005). Evaluation of the program to eliminate lymphatic filariasis in Vanuatu following 2 years of mass drug administration implementation: results and methodologic approach. Am J Trop Med Hyg.

[15] VBDCU (1998). Filariasis Screening Survey in Vanuatu, September 1997 to September 1998. Data Book. Department of Health.

[16] VBDCU (2013). Program data from the Vanuatu National Filariasis Control Programme: Draft dossier. Department of Health.

[17] Vanuatu-Government (2013). Vanuatu malaria programme performance review 2013.

[18] WHO. Global Programme to Eliminate Lymphatic Filariasis. Monitoring and epidemiological assessment of mass drug administration in the Global Programme to Eliminate Lymphatic Filariasis: a manual for national elimination programmes. Geneva: World Health Organization; 2011. [http://www.who.int/lymphatic_filariasis/resources/9789241501484/en/].

[19] UNICEF (2006). 2006 Multiple Indicator Cluster Survey (MICS). UNICEF and Malawi National Statistics Office.

[20] Brabin L (1990). Sex differentials in susceptibility to lymphatic filariasis and implications for maternal child immunity. Epidemiol Infect.

[21] Huppatz C, Capuano C, Palmer K, Kelly PM, Durrheim DN (2009). Lessons from the Pacific programme to eliminate lymphatic filariasis: a case study of 5 countries. BMC Infect Dis.

[22] Lemoine JF, Desormeaux AM, Monestime F, Fayette CR, Desir L, Direny AN, Carciunoiu S, Miller L, Knipes A, Lammie P (2016). Controlling Neglected Tropical Diseases (NTDs) in Haiti: implementation strategies and evidence of their success. PLoS Negl Trop Dis.

